# Next-Generation In Vitro Pulmonary Platforms for Respiratory Disease Modelling and Therapeutic Development: Current Advances and Future Prospects

**DOI:** 10.3390/medicina62050859

**Published:** 2026-04-30

**Authors:** Fariya Khan, Pratibha Verma, Aditya Singh, Manoj Kumar, Jalaj Gupta, Girijesh Kumar Patel, Samradhi Singh, Vinod Kumar, Alok Kumar Yadav, Vinod Verma

**Affiliations:** 1 Stem Cell Research Centre, Department of Hematology, Sanjay Gandhi Post Graduate Institute of Medical Sciences, Lucknow 226014, India; 2 ICMR-National Institute for Research in Environmental Health (NIREH), Bhopal 462030, India; 3 Department of Biotechnology, Motilal Nehru National Institute of Technology Allahabad, Prayagraj 211004, India; 4 Biotechnology Research and Innovation Council-National Institute of Animal Biotechnology (NIAB), Hyderabad 500032, India

**Keywords:** pulmonary diseases, in vitro models, 3D organoids, computational modelling, artificial intelligence

## Abstract

Pulmonary diseases such as Chronic obstructive pulmonary disease (COPD), asthma, pulmonary fibrosis, and acute respiratory infections remain a major global health challenge due to their complex pathophysiology and limited therapeutic options. Conventional 2D cultures and animal models have provided foundational insights; however, they often fail to accurately replicate the human lung’s intricate architecture, immune interactions, and patient-specific variability. Recent advances in vitro technologies have transformed pulmonary research, enabling the generation of physiologically relevant and translational disease models. The review highlights the progression of lung research platforms from traditional monolayer cultures to advanced systems such as air–liquid interface models and 3D lung organoids. These cutting-edge models more effectively mimic the biochemical, mechanical, and spatial microenvironment of the respiratory system, enhancing the fidelity of disease modelling and drug screening. In parallel, the integration of computational modelling and artificial intelligence (AI) has emerged as a powerful synergistic approach. AI-driven analytics facilitate high-throughput imaging, biomarker discovery, and patient-stratified therapeutic prediction, while computational tools simulate disease networks, mechanobiological interactions, and pharmacological responses. The convergence of these technologies supports a deeper understanding of pulmonary disease progression and accelerates the development of precision therapeutics. Collectively, this review underscores the transformative potential of combining in vitro lung models with advanced computational and AI methodologies. This synergy not only improves translational relevance and reduces reliance on animal testing but also paves the way for personalised interventions that better address the complexity of human pulmonary disease.

## 1. Introduction

Chronic lung diseases, including asthma, chronic obstructive pulmonary disease (COPD), lung fibrosis, pulmonary hypertension, and lung cancer, are major contributors to global healthcare burdens [1]. These conditions often arise from structural alterations and cellular damage caused by a combination of factors, such as long-term exposure to environmental elements such as air pollution, cigarette smoke, infectious agents, and genetic susceptibility [2]. The rising prevalence and disease burden of respiratory diseases worldwide can be attributed to increased exposure to these risk factors, along with the ageing of populations. The recent global coronavirus disease 2019 (COVID-19) pandemic, caused by severe acute respiratory syndrome coronavirus-2 (SARS-CoV-2), has been one of the major global crisis with over 317 million confirmed cases and 5.51 million deaths. Additionally, catastrophic wildfires worldwide around the world have added to the amplified exposure to hazardous air pollution levels exacerbating pulmonary issues.

Pulmonary diseases are often characterized by excessive mucus production and decreased mucociliary clearance [3,4], aberrant airway remodelling, pulmonary vasculature, and distal parenchymal tissue [5]. Subsequently, airflow limitation and alveolar tissue destruction result in the loss of lung function [3,4,5]. Although localized repair and regeneration in the lung are continuously facilitated by resident progenitor cells still their functionality may be compromised in disease conditions [6]. Consequently, lung transplantation remains to be the only definitive treatment for most end-stage lung diseases [7].

Advancing therapeutics for chronic lung diseases relies heavily on a comprehensive understanding of the underlying disease mechanisms. Despite considerable progress in research, the pathogenesis and progression of most pulmonary diseases remain poorly understood [8]. Addressing the rising global burden of respiratory diseases requires urgent development of rapid and efficient preclinical evaluation models to accelerate the production of therapeutics and vaccines with higher efficacy [9,10]. Traditional two-dimensional (2D) in vitro cell culture techniques have significantly to the existing knowledge of cellular behavior and dynamics, but they lack essential cell–cell and cell–extracellular matrix (ECM) interactions that are delineated to play crucial roles in cell signaling and function [11]. The application of bioengineering and computational tools and techniques has driven the development of innovative in vitro systems for modeling diseases in pulmonary research. Therefore, three-dimensional (3D) models comprising one or more matrix components and multiple cell types in amalgamation with high-end computational tools are emerging as the preferred standard for in vitro studies. The use of these models emulating pulmonary diseases will not only provide insight into disease mechanisms but also elevate the drug discovery and development process, along with its safety testing.

Drug development is a highly complex, time-consuming, and costly process, with clinical trials exhibiting high attrition rates [12]. On average, the research and development of a new drug approximately costs $2.5 billion, and it can take up to 15 years to be available on the market [13]. Despite significant collaborative efforts and rising expenditure only a limited number of drugs successfully enter into clinical trials [14]. In comparison fields such as cardiovascular or neurological, and pulmonary medicine have far fewer approved drugs and even fewer candidates in the pipeline to address the growing healthcare burden [15]. It is crucial to identify and eliminate ineffective drug candidates at the earliest to improve efficiency, reduce costs, and increase success rates in drug development. This can be achieved by employing advanced preclinical screening models that offer higher accuracy and reliability [13].

Laboratory-based in vitro models for preclinical studies that utilize various cell types and cell-based assays are commonly utilized to evaluate candidate drugs by the pharmaceutical industry and research laboratories [16,17]. While these models are straightforward, easy to handle, and provide high throughput for basic screening and testing of drugs, they fail to replicate the complex in vivo tissue architecture, physiology, and cellular interactions found in human cells and tissues. As a result, they are unable to predict drug metabolism processes and potential side effects [18]. Several animal models have also been employed to uncover the pulmonary conditions [19,20,21] but a major limitation is the significant differences in airway branching, cellular distribution, and lung tissue microstructures, as well as immune system functions, between humans and widely used rodents [22,23,24]. Rodent models used for COPD modelling, for instance, are limited by the mild phenotype, inconsistent response to cigarette smoke, and the lack of robust airway disease, making them less reflective of the processes involved in chronic bronchitis. Given these limitations, it is essential to recognize the challenges posed by animal models in pulmonary research [25]. Consequently, there is an urgent need for advanced human-relevant models that better replicate the human lung microenvironment, including tissue–tissue interfaces to enhance the modeling of respiratory diseases and accelerate pulmonary preclinical research, Thus, it is crucial to acknowledge and take into account the limitations of animal models for respiratory research.

Therefore, models should possess higher complexity to serve as a preclinical model that will allow for the translation of the experimental outcome from bench to bedside. Current models, such as human lung organoids, which represent a miniature form of lung tissue, have the potential to model pulmonary diseases with more precision. These advanced human-relevant models that mimic the human lung microenvironment, along with tissue–tissue interfaces, chemical gradients, and mechanical factors, are urgently required to better model respiratory diseases and expedite pulmonary preclinical research.

Unlike existing reviews that predominantly provide descriptive summaries of individual pulmonary in vitro models, this article introduces a conceptual and integrative framework that systematically organizes these models into a translational hierarchy based on their structural complexity, physiological relevance, and predictive capacity for clinical outcomes. This approach moves beyond conventional categorization by enabling a comparative evaluation of model systems in terms of their suitability for specific research and therapeutic objectives. Furthermore, this review uniquely incorporates artificial intelligence (AI) and computational modelling as functional components within this hierarchy.

**Figure 1:**
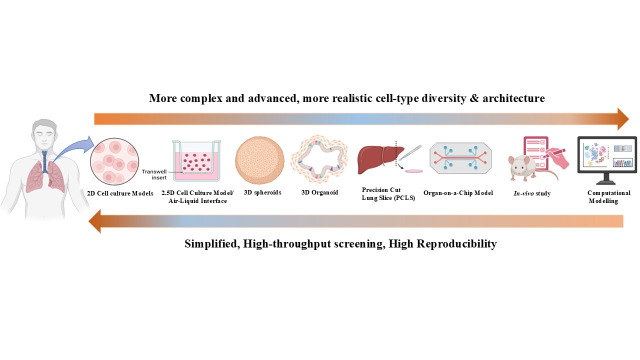
A conceptual schematic illustrating the translational hierarchy of pulmonary model systems.

Further, the article examines the recent advancements in the existing computational lung models and the application of AI, focusing on how these models integrate data with existing knowledge of lung structure to simulate and predict lung function in pulmonary disease. This review organizes advanced pulmonary model systems along a translational hierarchy that reflects their increasing biological complexity and predictive relevance for therapeutic discovery (Figure 1).

## 2. Translational Hierarchy of In Vitro Pulmonary Model Systems

At the foundational level, two-dimensional (2D) and 2.5D culture systems remain indispensable for mechanistic investigations, enabling detailed analyses of cellular signalling pathways, genetic regulation, and high-throughput pharmacological screening. Building upon these systems, intermediate-complexity models, including spheroids and multicellular 3D cultures, provide improved representation of cell–cell interactions and microenvironmental dynamics that influence tissue organization and disease progression. Further along this hierarchy, high-fidelity disease models such as lung organoids and organ-on-a-chip more closely recapitulate the structural architecture, cellular heterogeneity, and functional properties of native lung tissues, making them particularly valuable for modelling patient-specific disease phenotypes and therapeutic responses. Complementing these experimental platforms, computational modelling and artificial intelligence-driven analytical frameworks are increasingly being integrated to interpret complex multi-omics datasets, identify predictive biomarkers, and enhance drug response prediction. In line with the proposed translational hierarchy of pulmonary models, Table 1 summarized a comparative evaluation of commonly used in vitro platforms, enabling a clearer understanding of their relative applicability across mechanistic studies, disease modelling, and therapeutic screening.

## 3. Two-Dimensional (2D) In Vitro Models for Pulmonary Disease

In vitro lung models play a crucial role as an accurate and reliable tool for studying lung biology and disease, bridging the gap between multiple disciplinary boundaries. Pulmonary diseases like non-small cell lung cancer or other lung malignancies to respiratory infections, such as influenza or COVID-19, create a significant burden on both patients as well as the healthcare system. Leveraging in vitro models to deepen our understanding of these diseases is essential for advancing the development of life-saving therapies with higher potential and efficacy.

Researchers are widely utilizing in vitro models for pulmonary diseases to gain deeper insight into diseases like chronic obstructive pulmonary disease, lung cancer, COVID-19, and asthma. These models serve as valuable tools for studying disease mechanisms, identifying biomarkers, and evaluating the effects of potential treatments [26]. Additionally, in vitro lung models play a crucial role in understanding the pathogenic mechanisms of emerging airborne viruses, such as SARS-CoV-2, and in developing innovative prevention strategies and therapies for respiratory infections that could escalate into epidemics or pandemics [27].

The two-dimensional (2D) cell cultures are the most commonly used in vitro model systems for studying pulmonary diseases. This model typically consists of key cell types involved in the pathogenesis of respiratory diseases, such as fibroblasts, alveolar epithelial cells, bronchial epithelial cells, and small airway epithelial cells that are derived from healthy or diseased subjects or are commercially available [28] (Table 1).

A fibroblast reporter cell line (10-4ABFP) with α-smooth muscle actin (SMA) promoter, a marker of the myofibroblast, was recently developed to study the progression of pulmonary fibrosis [29]. Numerous in vitro studies have focussed on exploring the effects of profibrotic or antifibrotic molecules on cultured fibroblast function and the resulting phenotype. For example, biomolecules such as IL-6 and fibroblast growth factors (FGF)9 and FGF18 have been shown to promote apoptosis resistance [30,31] and enhance migration and invasion capacities in fibroblasts derived from IPF patients [31].

Bronchial fibroblasts isolated from healthy and asthma patients were used in a study that identified IL-13’s association with the pathological involvement of the histone demethylase JMJD2B/KDM4B, which plays a significant role in the development of steroid-resistant asthma [32]. The COPD patients derived fibroblasts exhibit a secretory phenotype marked by the upregulation of various inflammatory markers, as well as matrix proteoglycans versican and elastin [33]. Supporting models that implicate inflammatory fibroblasts in proximal lung regions as drivers of lymphocytic inflammation and emphysema, a recent study reported that conditional deletion of Hedgehog-interacting protein (*HHIP*) *Hhip* in Gli1^+^ fibroblasts resulted in tissue-resident T cell expansion and epithelial cell loss [34].

In another study by Pedroza et al., 2015 [35], during in vitro experiments using A549, a human AEC line, and MLE-12 (murine lung epithelial-12), an immortalized murine AEC line demonstrated that activation of STAT-3 by TGF-β–plays a key role in promoting the expression of genes associated with injury and mesenchymal in AECs. Additionally, it also influenced the differentiation of lung fibroblasts into myofibroblasts. Similarly, the hBECs and Beas-2B cells were utilized in a study by Wen et al., 2019 [36], the findings from this research demonstrated that the silencing FUNDC1 effectively suppressed CSE-induced mitochondrial autophagy and apoptosis, thereby mitigating the progression of COPD. The results were supported by in vivo data as well. This effect was mediated through the interaction of FUNDC1 with DRP1, which suggests that FUNDC1-DRP1 interaction could serve as a potential therapeutic target for COPD treatment.

Alveolar epithelial cell cultures play a crucial role in understanding the initiation and progression of pulmonary diseases, especially IPF. A549 cells, a human AT2 cell line, is often used as submerged 2D cultures, although it does not fully mimic the pulmonary epithelium; still, they are commonly employed to investigate TGF-β1-induced EMT. This process is characterized by the downregulation of epithelial markers such as E-cadherin and CK19, along with enhanced expression of vimentin, fibronectin, and elevated collagen production [37]. In a very recent study conducted in 2023, cryopreserved type 2 AEC (AEC2) samples from both healthy lungs and COPD-afflicted lungs were cultured in decellularized healthy human lung slices to study the plasticity of their ECM production. Healthy AEC2, treated with TGF-β1, showed a stable AEC marker profile and demonstrated changes in ECM production, particularly interstitial ECM production, without losing AEC-specific markers. COPD-derived AECs exhibited similar proliferation as healthy AECs with few differences in gene and protein expression but maintained higher levels of the disease marker HLA-A. This study highlights the previously unexplored role of AEC in ECM turnover and suggests that AEC may contribute directly to pathological lung remodeling [38].

Traditional 2D culture offers several practical and logistical advantages, especially compared to in vivo models, such as scalability, suitability for cell imaging, low cost, and easier experimental data interpretation [39,40]. However, in highly complex organs like the lung, where intercellular communication and mechanosensing are difficult to replicate artificially, traditional 2D cell culture have clear limitations. Also, research comparing cellular responses in 2D versus 3D environments shows that cells in 2D cultures often exhibit physiologically irrelevant behavior, relying more on cell–substrate interactions than cell–cell and cell-ECM communication. These cells frequently display altered morphologies, abnormal proliferation rates, and different gene and protein expression profiles compared to those in vivo [39,40]. While the 2D cell cultures lack the intricate tissue architecture found in vivo, they offer significant advantages as well, such as low cost and maintenance time with higher reproducibility, along with simpler scalability with easy functional analysis making them valuable for high-throughput assays [41]. However, recent years have highlighted the limitations of these models, particularly their lack of complexity. Studies conducted on 2D monolayers often overemphasize the effects of therapeutic agents, which frequently fail to replicate in in vivo studies as they lack the complex cellular communication networks found in three-dimensional (3D) cultures, which more effectively mimic the conditions and interactions present in vivo (Table 2).

## 4. 2.5D In Vitro Models for Pulmonary Disease

To address the shortcomings, 2D culture methods can be improved to mimic the native tissue microenvironment better. Enhancements such as dynamic culture conditions [72] or surface modifications with physical micropatterns have influenced cell morphology, attachment, and migration, leading to the concept of 2.5D cell culture. The advent of new culture platforms to better mimic the 3D lung microenvironment has resulted in the generation of 2.5D models [73,74]. The 2.5D cell culture may represent an important compromise between scalability/reproducibility and representativeness of the in vivo cell microenvironment, serving as relatively simple approaches to achieve consistent and physiologically relevant cellular responses. Air-interfaced cultures are most appropriate in this context, with the supply of nutrients both apically and basally during differentiation, and airlifting post-differentiation, reflecting the environments and processes found in vivo. Additionally, ALI culture grown in a Transwell configuration provides a more in-depth analysis of viral entrance. For example, it is possible to inoculate with infected serum or use aerosol deposition atop the cell culture, mimicking entrance in vivo. Collecting cell supernatant both apically and basally then permits the study and spatial identification of cell-specific entrance with methods such as 3D immunofluorescence rendering and quantification as well as RNA extraction, viral plaque forming assays, and scanning electron microscopy [75]. Culturing primary cells in Transwell configurations and the formation of a pseudostratified epithelium permits patient- and disease-specific studies of response to infection and therapeutics in 2.5D. Multiple cell types, such as epithelial, endothelial, or immune cells can also be co-cultured on either the basal or apical side on the Transwell filter insert, representing a more complex and complete model. Additionally, functional confocal microscopy and live capture video analysis can obtain pathogen-induced measures of immune cell recruitment, receptor entrance, and transmigration through the membrane and cell layers [76].

Cell culture has become an essential tool for investigating the fundamental biophysical and molecular mechanisms by which cells contribute to tissue and organ generation, as well as their function, and their alteration when affected by disease. 2.5D culture improve upon classical 2D monolayers but still has important limitations when modelling lung physiology and disease. 2.5D cultures provide partial ECM interaction but still lack the fully embedded 3D architecture present in lung tissues such as alveoli or airway epithelium. Consequently, spatial cell–cell and cell–matrix interaction are not fully reproduced [77]. Cells typically interact with ECM only on one side, while the opposite surface faces the culture medium, which does not reflect the in vivo environment. Also, the large volume of culture medium, causes dilution of locally secreted cytokines, growth factors, and ECM components that normally regulates lung cell behavior. This can disrupt signalling involved in epithelial repair, inflammation, and fibrosis pathways.

## 5. Three-Dimensional (3D) In Vitro Models for Pulmonary Disease

Two-dimensional culture-based model systems have long been the standard in pulmonary disease research; however, recent developments have increasingly shifted focus towards 3D models due to their ability to more accurately replicate the biochemical and biomechanical microenvironments of the living tissues [78]. Given the limitations of 2D cell culture models and their restricted applicability for extrapolating data, 3D models have emerged as superior platforms for cell- and organ-based assays. These models facilitate a more comprehensive evaluation of disease mechanisms using patient-derived cells and support the safety assessment of low-clearance drugs and studies involving multiple dosing [79]. Three-dimensional cell culture models exist in diverse forms, ranging from simple spheroids to more complex structures like organoids and organs-on-chips [80].

### 5.1. Spheroids

Spheroids represent one of the earliest forms of 3D culture systems used in pulmonary disease modeling. These spherical aggregates of cells grow in layered arrangements, creating a three-dimensional geometry. Compared to traditional 2D systems, spheroids offer a significant advantage by replicating physiologically relevant cell–cell and cell–extracellular matrix (ECM) interactions. Initially developed for modeling malignancy progression in vitro, they are often derived from patient tumor samples or immortalized cancerous cell lines. Spheroids can be generated using either scaffold-based or scaffold-free approaches, with the latter being the more commonly employed approach. Their mechanical stability primarily relies on the cytoskeleton of the cells. Spheroids can be generated using various methods, which include pellet culture methods, hanging drop methods, use of ultra-low attachment or low adherent plates and bioreactors at larger scales [77].

In a recent study by Miranda et al., 2024 [81], 3D lung tissue spheroids were utilized as a model for SARS-CoV-2 infection and drug screening. These spheroids were generated by coculturing human alveolar or bronchial epithelial cells with human lung stromal cells, followed by infection with SARS-CoV-2. The study highlighted that coculture spheroids of epithelial and stromal cells could serve as a cost-effective model for studying infection model for SARS-CoV-2 infection. Furthermore, the authors proposed using these 3D spheroid platforms for drug screening to investigate potential treatments targeting cytokines release during viral infection.

Similarly, to better replicate the in vivo environment and study the effect of air pollutants such as cigarette smoke on lung cells, a known contributor to diseases like COPD and asthma, Baarsma et al., 2022 [82], cultured the bronchial epithelial cells in a 3D environment. Treatment of these epithelial spheroids with TGF-β led to a reduction in E-cadherin and an increase in collagen I expression, suggesting the activation of epithelial-to-mesenchymal transition (EMT). Similarly, exposure of spheroids to diesel exhaust particles (DEP) induced an EMT-like phenotype. Collectively, their findings demonstrated that air pollutants could trigger an EMT-like phenotype of BEAS-2B cells cultured in 3D spheroid models. This approach provides a promising platform for developing therapies targeting pulmonary diseases caused by pollutants.

In another study by Xu et al., 2024 [83], 3D spheroids of primary human lung fibroblasts (HLFs) were used to investigate the effect of PM2.5 on cellular senescence. Compared to traditional 2D cultures, 3D spheroids exhibited greater sensitivity to PM2.5-induced cytotoxicity. PM2.5 exposure led to enhanced senescence-associated β-galactosidase (SA-β-gal) activity, DNA damage, increased p21 expression, suppressed cell proliferation, and a heightened inflammatory response. These effects were linked to the activation of the TGF-β/Smad3 axis and HMGB1 pathway. The findings highlight the potential of 3D models to better mimic in vivo-like conditions and provide new insights into PM2.5 toxicity.

Despite their significant advantages, spheroid cultures face several challenges, such as difficulties in achieving uniform spheroid size and maintaining precise cell ratios in co-culture. Also, since they are mostly derived from cell lines [84,85,86] or primary patient-derived cells [87,88], they are deprived of stem or progenitor cells that have the property to self-renew and differentiate, thereby making it difficult to generate complex and multicellular structures. These limitations have prompted the generation of more advanced 3D in vitro systems, such as organoids. Organoids are specialized culture systems that enable organ-specific progenitor cells to expand rapidly and form a 3D structure composed of cell types unique to the organ being studied [89]. These systems provide an effective solution for modeling the complexities of lung biology, as they can be developed using a variety of cell types, including airway secretory cells, airway epithelial basal progenitor cells, and type 2 alveolar epithelial cells [90].

### 5.2. Lung Organoids (LOs)

Researchers have introduced lung tissue organoids in lung studies to address the constraints of traditional 2D cultures and cross-species models. These organoids, often called “organs in a dish,” strive to replicate essential elements of tissue structure seen in living organisms. They combine various cell types from the same species to create a microenvironment that mimics the organ’s function [91].

**Figure 2:**
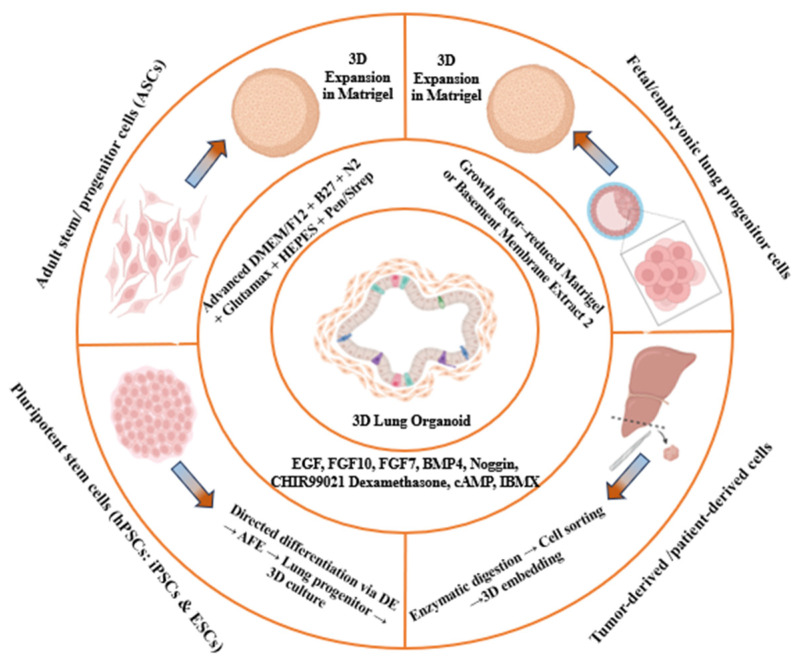
Different cellular sources used to generate 3D lung organoids.

Human lung organoids (LOs) can be generated using various methods and biological materials, each with its unique advantages and drawbacks. One common approach involves utilizing induced pluripotent stem cells (iPSCs), reprogrammed adult cells capable of differentiating into various cell types found in the lung. iPSC-derived lung organoids offer the advantage of patient-specific modeling and disease understanding [92]. Another method involves isolating and culturing primary lung epithelial cells from biopsies or organ donors [93]. These primary cell-derived organoids provide a more physiologically relevant model of the lung but may be limited by the availability of donor tissue. Additionally, lung organoids can be generated from lung tissue-resident stem cells, such as basal cells or bronchioalveolar stem cells, which possess self-renewal and differentiation capabilities [94]. Furthermore, advancements in gene editing technologies, such as CRISPR-Cas9, have enabled the precise manipulation of organoid genomes to study genetic contributions to lung diseases [95]. Each method and cell source offers unique advantages and challenges, contributing to the versatility and utility of lung organoid models in research and medicine. Choosing the most suitable approach for a specific application is crucial. Irrespective of the cell source, a fundamental step shared among all protocols for organoid formation involves embedding cells in an extracellular matrix, which acts as the foundation for tissue culture and supports the development of the 3D architecture [96]. Once fully differentiated, organoids can be propagated, expanded, and utilized for various purposes including basic tissue research [97], studies on cell interactions [98], and testing of cancer drugs [99,100,101] (Figure 2).

#### 5.2.1. Lung Organoids from Fetal Lung Cells

The early stages of 3D lung cell cultures, termed organoids or “mass cultures,” are initiated with a blend of cells sourced from digested fetal lung tissue, comprising epithelial, endothelial, mesenchymal, and hematopoietic cells [102]. These cells were cultivated at the air–liquid interface (ALI) on a floating membrane filter, facilitating their differentiation into mature ATII cells and the formation of connective tissue. Despite these advancements, these initial organoid cultures encountered limitations, including inadequate 3D growth and the absence of supportive structures, leading to the formation of heterogeneous cell aggregates [103]. Moreover, the culture duration was constrained due to overgrowth of mesenchymal cells.

These shortcomings were addressed by the scientists and with advancements, scientists have begun utilizing fetal-derived lung organoids (LOs) to investigate the underlying mechanisms of various lung diseases. A notable study by Lim et al. (2023) [104] introduced a novel approach using human fetal lung-derived alveolar organoids to uncover the mechanisms governing surfactant protein C maturation, which holds relevance to interstitial lung disease [105]. Additionally, researchers have embarked on studies involving organoid modeling of human fetal lung alveolar development, aiming to elucidate the intricate mechanisms of cell fate patterning and their implications in neonatal respiratory diseases. These endeavors signify a significant stride towards better understanding the pathophysiology of lung disorders and potentially paving the way for novel therapeutic interventions.

Fetal lung cell-derived organoids offer a unique opportunity to delve into the intricate mechanisms underlying COPD pathology. These organoids, derived from fetal lung cells, provide a platform to study early developmental processes and how they may influence COPD susceptibility later in life. By examining the differentiation patterns, cellular interactions, and inflammatory responses within these organoids, researchers can gain valuable insights into the initial stages of COPD development. Moreover, the ability to manipulate genetic and environmental factors in these organoids allows for the exploration of specific disease triggers and risk factors. Ultimately, leveraging these organoids in COPD studies holds promise for uncovering novel molecular pathways and advancing precision medicine approaches for this complex respiratory disease.

Fetal-origin lung organoids have provided valuable insights into early lung development; however, several limitations restrict their broader translational application. One major constraint is their developmental stage bias, as organoids derived from fetal tissue largely resemble immature lung epithelium and therefore may not accurately represent the cellular composition, physiology, or disease mechanisms of the adult lung. Additionally, the limited availability of fetal tissue and donor-to-donor variability make it difficult to standardize experiments and scale studies for high-throughput applications such as drug screening. Furthermore, the use of fetal tissue raises significant ethical and regulatory concerns, as procurement typically involves tissue obtained following elective pregnancy termination, requiring strict ethical oversight, informed consent procedures, and compliance with institutional and national regulations. These ethical constraints, along with societal and policy considerations, can limit tissue accessibility and restrict widespread adoption of fetal-derived organoid systems in lung research [106].

#### 5.2.2. Lung Organoids from Primary Lung Cells

Basal cells isolated and expanded from human lungs have been instrumental in generating organoids, known as tracheospheres or bronchospheres, depending on their origin from the trachea or large airways, respectively. These organoids typically consist of TRP63+ KRT5+ basal cells, along with functional multiciliated cells and secretory goblet cells expressing MUC5AC and MUC5B [107]. Given that basal cells also reside in the nasal epithelium, the prospect of deriving organoids, termed “nasospheres,” from these cells presents a convenient method for patient-specific organoid generation, particularly for drug screening purposes [108]. Notably, organoids derived from human basal cells have been utilized to screen for cytokines and other proteins influencing the balance between ciliated and secretory cells. This research avenue holds promise for identifying potential therapeutic targets for disorders like COPD, where such balance disruptions are implicated in disease pathology.

Besides basal cells, alveolar cells are also a common type of cells that are exploited for the generation of organoids, as the clinical significance of respiratory disorders, such as COPD and idiopathic pulmonary fibrosis, underscores the urgent need for a deeper understanding of the fundamental biology underlying alveolar stem cells and their microenvironment, or niche. Currently, there is limited knowledge regarding the potential heterogeneity within the population of alveolar epithelial type 2 cells (AEC2s) and whether distinct subsets, particularly those located at the lung periphery and pleural surface, exhibit heightened proliferative and regenerative capacities compared to their counterparts in the lung interior [108]. Furthermore, there is a dearth of understanding concerning the intricate molecular crosstalk between the alveolar epithelium and neighbouring mesenchymal, endothelial, and immune cell populations within the alveolar region. Crucially, exploring whether small molecules and therapeutic agents can mitigate or reverse pathological alterations once they have occurred represents a pressing avenue of investigation. These critical questions and more are being actively pursued through organoid cultures, wherein AEC2s, in conjunction with supportive cell types, are manipulated to both proliferate and differentiate into AEC1s, offering promising insights into potential therapeutic strategies and avenues for intervention for diseases where the integrity of these cells is compromised.

#### 5.2.3. Lung Organoids from Induced Pluripotent Stem Cells

Human iPSC-derived lung organoids generation represents a major advance in pulmonary disease modeling, drug screening, and regenerative medicine. Optimization of culture media with specific growth factors, murine and human iPSCs are first directed toward definitive endoderm, followed by sequential generation of anterior foregut endoderm (AFE), ventral anterior foregut endoderm cells (VAFECs), and, lastly, NKX2-1^+^ lung progenitors [106,109,110]. The resulting organoid models faithfully mimic the native lung tissue architecture and microenvironment, enabling mechanistic pulmonary disease pathogenesis investigations, pharmacological screening, and precision medicine. Owing to their multicellular complexity, and ability to capture intercellular interaction, these lung organoids have been used to model a range of pulmonary conditions, including cystic fibrosis (CF), idiopathic pulmonary fibrosis (IPF), and respiratory viral infections such as SARS-CoV-2 [111].

Emerging evidence supports the application of iPSC-derived lung organoids in elucidating disease-specific phenotypes and evaluating therapeutic interventions. Wilknson et al. [112] used human fetal-derived mesenchymal organoids for IPF modelling, and high α-SMA and collagen I expression were observed post-treatment with TGF-β for 2 days, which are stringent markers of fibrosis [113]. In another study by Ptasinski et al., hiPSCs derived alveolar epithelial organoids were used to model alveolar epithelial injury in IPF using a fibrosis cocktail. Upon treatment with FC, IPF-related changes were observed, such as production of extracellular matrix, and induction of cellular senescence-associated genes, epithelial injury and reprogramming (e.g., loss of SFTPC expression and induced VIM expression). Also, the FC treatment elevated the expression of KRT17 and KRT8, both recently identified in single-cell RNA-Seq studies as markers of dysregulated epithelial repair in IPF [114].

In another study by Chan et al. (2022) [115], nasopharyngeal and bronchial organoids were established from healthy individuals and those with COPD to recapitulate the disease at the individual level. They observed that, in contrast to healthy organoids, reduced ciliary beat frequency and goblet cell hyperplasia were observed in COPD organoids, which are the hallmark features of the disease. Also, altered cellular differentiation trajectories in COPD organoid were revealed by single-cell transcriptomics. The incorporation of CRISPR-Cas9 genome editing, single-cell transcriptomic profiling, and high-throughput drug screening, iPSC-derived lung organoids will aid in the advancement of translational research and bridge the gap between in vitro models and clinical applications. Also, the increasing complexity of data generated from these advanced model systems necessitates the use of computational modelling for meaningful interpretation. The integration of computational models is essential to translated organoid-derived experimental data into actionable insights and therapeutic strategies.

## 6. Integration of Computational Tools in Pulmonary Disease Modelling

The need for improved diagnosis and assessment of pulmonary diseases in order to elucidate the pathophysiology behind such diseases is the driving force for integrating multiscale computational modelling with the different model systems available [116]. The large datasets derived from the experimental analysis of in vitro models, such as multiomics datasets, can be analysed by incorporating advanced algorithms and simulations, which also aid researchers in creating real-time representations of lung dynamics and function. Additionally, integrating medical imaging data, such as CT scans and MRIs, allows researchers to incorporate lung structures with more accuracy. As a result, computational modelling holds the potential to facilitate experimental strategies, optimise our discoveries, and ultimately lead to better clinical outcomes for individuals affected by lung diseases [117].

Computational modelling offers a powerful approach for investigating this relationship between imaging measurements and disease severity, as well as for understanding the effects of different disease subtypes, key to developing improved diagnostic methods [116]. Computational tools play an essential role in advancing our understanding of pulmonary diseases using in vitro models. These models serve as a powerful experimental system that mimics the structure and function of lung tissue, and computational approaches help enhance their utility by enabling deeper analysis, modelling, and predictions.

To enhance the lung-specific relevance of computational modelling, recent studies have increasingly focused on integrating data derived from advanced pulmonary in vitro systems, including lung organoids and air–liquid interface cultures. For instance, transcriptomics and single-cell RNA sequencing datasets generated from human lung organoids and air–liquid interface cultures have been used to construct disease-specific regulatory networks in conditions such as IPF and COPD [118]. These models enable the identification of key signalling hubs, including the TGF-β, Wnt/β-catenin, and Notch pathways, which are critically involved in epithelial injury, fibroblast activation and aberrant tissue remodelling.

Moreover, computational frameworks have been applied to simulate epithelial–mesenchymal crosstalk within lung organoids, allowing researchers to predict how perturbations in specific pathways influence disease progression. Such lung-specific modelling approaches provide a more accurate representation of the pulmonary microenvironment compared to generalized systems and significantly improve the translational applicability of in vitro findings.

### 6.1. Data Interpretation and Analysis

The advances in vitro models, such as 3D organoids and organ-on-a-chip, generate large and complex datasets from multiple omics layers, such as transcriptomics, proteomics, and metabolomics. Each of these layers captures distinct yet interconnected aspects of cellular biology. Together, these layers form a comprehensive representation of biological activity, but their integration poses significant computational challenges due to differences in data types, scales, and underlying biological processes. Advanced computational approaches, such as machine learning algorithms, dimensionality reduction techniques (e.g., principal component analysis or PCA), and network-based methods, are employed to integrate these datasets effectively [119]. By harmonizing multi-omics data, researchers can construct interaction networks and identify key regulatory genes or pathways responsible for specific cellular behaviors or disease mechanisms. For example, network analysis may uncover central hubs in signalling pathways or transcription factors driving disease progression, offering insights into potential therapeutic targets. This integrated systems-level approach not only deepens the understanding of lung biology but also enables the discovery of novel biomarkers and interventions for lung diseases, demonstrating the transformative potential of multi-omics in pulmonary disease research [120]. The sheer volume and complexity of these data necessitate robust computational pipelines for effective processing and analysis. These pipelines employ a combination of statistical methods, bioinformatics tools, and machine learning algorithms to identify meaningful trends and patterns.

In pulmonary research, high-throughput data analysis has enabled significant advancements. For example, by profiling gene expression changes in lung organoids exposed to toxins, researchers can identify molecular signatures of injury and repair. Similarly, imaging analyses of organoids affected by viral infections, such as SARS-CoV-2, have revealed structural and functional disruptions mimicking those observed in human lungs. These findings not only improve our understanding of disease mechanisms but also aid in the development of targeted therapeutic strategies and biomarkers for early diagnosis.

Moreover, these computational approaches are increasingly applied to lung-specific datasets derived from organoids and patient samples. Single-cell RNA sequencing of lung organoids exposed to fibrotic stimuli has enabled the disease progression [121]. Similarly, integration of multi-omics datasets from COPD patient-derived airway epithelial cultures has revealed dysregulated inflammatory and oxidative stress pathways. These lung-specific analyses not only improve mechanistic understanding but also enable the identification of clinically relevant biomarkers for respiratory diseases.

### 6.2. Disease Mechanism Exploration

Pathway modelling is a powerful computational approach used to simulate intracellular and intercellular signalling networks, shedding light on the molecular mechanisms driving pulmonary diseases such as pulmonary fibrosis and lung cancer. By building dynamic models of signalling pathways, researchers can explore how disruptions in molecular communication lead to pathological outcomes, including excessive tissue scarring, uncontrolled cell growth, or immune system dysregulation. These models are particularly valuable for dissecting complex biological processes and identifying potential therapeutic targets. Computational tools used in pathway modelling rely on quantitative data from multi-omics studies, such as transcriptomics and proteomics, to construct and simulate signalling networks [122].

For example, in pulmonary fibrosis, researchers can model growth factor signaling pathways, such as transforming growth factor-beta (TGF-β) and fibroblast growth factor (FGF) signaling, to understand how these pathways drive fibrosis. Dynamic simulations of these pathways can reveal the cascade of molecular events such as the activation of transcription factors, upregulation of extracellular matrix (ECM) production, and dysregulation of apoptosis that lead to excessive deposition of scar tissue and stiffening of lung tissue [123].

Similarly, in lung cancer, pathway modelling can simulate oncogenic signaling networks, such as the epidermal growth factor receptor (EGFR) or phosphoinositide 3-kinase (PI3K)/Akt pathways, to study how mutations or overactivation lead to uncontrolled cell proliferation and metastasis. These models allow researchers to predict the effects of specific mutations or pathway inhibitors, providing a foundation for targeted drug development. For instance, pathway modeling might reveal how blocking downstream effectors in the EGFR pathway could reduce tumor growth while minimizing side effects. Pathway modeling also facilitates the exploration of intercellular communication within the lung microenvironment. For example, by simulating crosstalk between epithelial cells, fibroblasts, and immune cells, researchers can investigate how inflammatory signaling contributes to fibrosis or tumor progression. These insights are invaluable for understanding disease dynamics and for identifying points of intervention that could disrupt pathogenic signaling while preserving normal cellular functions [124].

In pulmonary research, pathway modeling extends beyond mechanistic understanding. It enables the testing of hypotheses in silico, saving time and resources in experimental validation. Furthermore, these models can predict patient-specific responses to therapies based on molecular profiles, paving the way for precision medicine. By unraveling the complex signaling networks that underpin diseases like pulmonary fibrosis and lung cancer, pathway modeling serves as a cornerstone of modern computational biology, offering new opportunities to combat these challenging conditions [125].

### 6.3. Drug Screening and Development

Virtual drug screening is a transformative approach in pulmonary research, leveraging computational tools to simulate interactions between therapeutic compounds and advanced in vitro models. This technique enables researchers to predict the efficacy, safety, and potential toxicity of drugs, offering a rapid and cost-effective alternative to traditional drug discovery methods [123]. By prioritising the most promising compounds for experimental testing, virtual screening significantly reduces resource consumption and accelerates the development of treatments for complex lung diseases such as pulmonary fibrosis, asthma, and lung cancer [126]. Using detailed molecular models and omics data from in vitro models, virtual screening platforms predict how candidate drugs interact with cellular targets, such as receptors, enzymes, or signaling molecules.

Beyond molecular interactions, virtual screening also incorporates pharmacokinetic and pharmacodynamic modeling to assess how drugs are absorbed, distributed, metabolized, and eliminated in the lung microenvironment [127]. This is particularly valuable in predicting localized drug effects, such as aerosolized therapies for conditions like chronic obstructive pulmonary disease (COPD) or acute respiratory distress syndrome (ARDS) [127]. Computational models can simulate how drugs penetrate airway tissues, interact with target cells, and potentially induce side effects, providing critical insights into both efficacy and safety.

This approach also facilitates the repurposing of existing drugs for pulmonary diseases. Computational tools can analyse drug libraries to identify compounds with unexpected efficacy against lung-specific targets, greatly expanding therapeutic options [128]. In a study by Tamang et al., (2025) [129], fosamprenavir and tirofiban were identified as promising hits that can exhibit potent macrophage metalloelastase inhibition which was also validated by the MD simulation and DFT-based calculations. This study not only revealed these repurposed drugs as effective macrophage metalloelastase inhibitors but also opened up a horizon in developing novel potent macrophage metalloelastase inhibitors for the management of cancer and COPD in the future.

**Figure 3:**
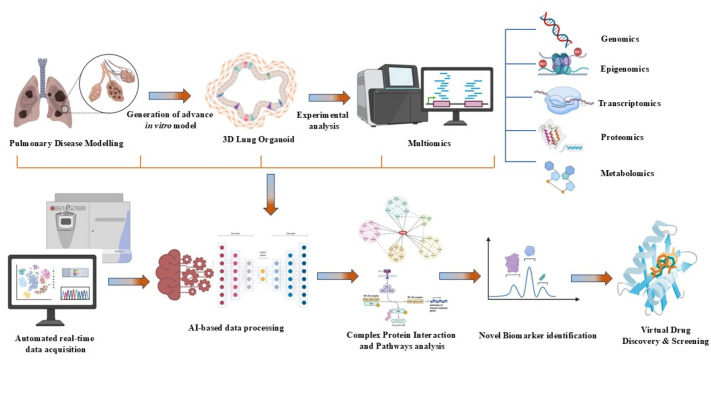
A synergistic pipeline for identifying therapeutic targets using an advanced in vitro lung model with AI-driven analytics for therapeutic target and drug discovery.

Moreover, by predicting potential off-target effects or toxicities, virtual screening helps researchers eliminate unsuitable candidates early in the development process, saving both time and costs. Virtual drug screening exemplifies the integration of computational power and biological systems in modern pulmonary research. By simulating drug-organ interactions in silico, this approach accelerates the identification and optimisation of treatments, ultimately improving the likelihood of clinical success and addressing unmet medical needs in lung health (Figure 3).

## 7. The Synergy of AI and In Vitro Models in Pulmonary Research

The integration of Artificial Intelligence (AI) with in vitro models has brought a paradigm shift in pulmonary research, offering unprecedented opportunities to study lung diseases with greater accuracy and efficiency. In vitro models, including 3D lung organoids, primary cell cultures, and cell-based assays, have long been invaluable in simulating lung biology and disease processes [130]. However, these models often generate vast, complex datasets that require advanced analytical techniques to fully harness their potential. AI, particularly machine learning (ML) and deep learning (DL), has emerged as a powerful tool to enhance the interpretation of these datasets, providing insights into disease mechanisms, drug responses, and potential therapeutic interventions [131].

AI’s ability to process large volumes of data is crucial in overcoming the inherent limitations of in vitro models. For example, 3D lung organoids mimic the structural complexity of the human lung, but their behavior can be influenced by numerous factors, such as gene expression, protein levels, and cellular interactions. AI algorithms can analyze these multifaceted datasets comprising transcriptomics, proteomics, metabolomics, and imaging data enabling researchers to identify patterns and correlations that might otherwise go unnoticed [132]. For instance, ML models can be trained to predict how changes in gene expression influence cellular behavior in models like lung organoids, facilitating the identification of key regulatory networks involved in diseases like pulmonary fibrosis or lung cancer. This ability to process and integrate multi-omics data enhances our understanding of lung disease at a systems level, providing a holistic view of disease progression and potential therapeutic targets [133]. AI also plays a significant role in automating the analysis of complex imaging data, such as that generated by high-resolution microscopy and confocal imaging of in vitro model systems [134]. In traditional settings, manual analysis of such images can be time-consuming and prone to human error. AI-driven image recognition and segmentation techniques, however, can efficiently process vast amounts of imaging data, identifying subtle structural and morphological changes [135]. For example, AI algorithms can detect early signs of fibrosis or tumor formation by analyzing the arrangement and behavior of cells and extracellular matrix components in lung organoids. This automation not only accelerates the research process but also increases the accuracy and reproducibility of results, which is crucial for drug discovery and disease modeling.

AI’s integration with in vitro models also supports personalized medicine in pulmonary research. By combining AI with patient-specific data, such as genomics or imaging, researchers can create individualized models that predict how a particular patient’s lung tissue will respond to different treatments [136]. These personalized models enable the development of tailored therapeutic strategies that are more likely to succeed in treating individual patients. For instance, in lung cancer research, AI can predict which drug combinations will be most effective based on the molecular profile of a patient’s tumor, providing a personalized approach to treatment that improves clinical outcomes [137].

Furthermore, AI models such as Sybil use deep neural networks to analyze low-dose CT scans and predict an individual’s future risk of lung cancer up to six years in advance from a single scan, without requiring additional clinical variables or manual radiologist annotations. Beyond imaging-based diagnostics, AI frameworks are also developed to model disease progression and guide therapeutic discovery. One such example is UNAGI, a deep generative neural-network platform that integrates time-series single-cell transcriptomic data to reconstruct cellular trajectories during disease progression and perform in silico drug perturbation analysis.

Moreover, AI’s ability to identify novel biomarkers through in vitro models accelerates the discovery of early diagnostic tools for lung diseases. By analyzing gene expression, protein profiles, and cellular behaviors, AI can pinpoint specific biomarkers associated with disease onset or progression, which can be used for early detection and monitoring of pulmonary conditions. This capability is particularly important in diseases like lung cancer, where early diagnosis is critical for improving survival rates. AI-driven biomarker discovery can also guide the development of companion diagnostics, helping clinicians select the most appropriate treatments for patients based on their molecular profiles [138].

AI-driven image analysis platforms have been successfully applied to respiratory organoids for the automated quantification of morphology, lumen formation, and structural heterogeneity, allowing high-throughput and unbiased phenotypic assessment [139]. In parallel, machine learning based approaches have been employed to quantify fibrosis severity in pulmonary models, improving the reproducibility and sensitivity of traditional histopathological scoring systems [140]. Importantly, the integration of AI with single-cell RNA sequencing datasets derived from lung tissues and organoid systems has enabled the identification of disease-relevant cellular subpopulations and regulatory pathways in conditions such as IPF, including translational epithelial states and activated fibroblast phenotypes [141].

**Figure 4:**
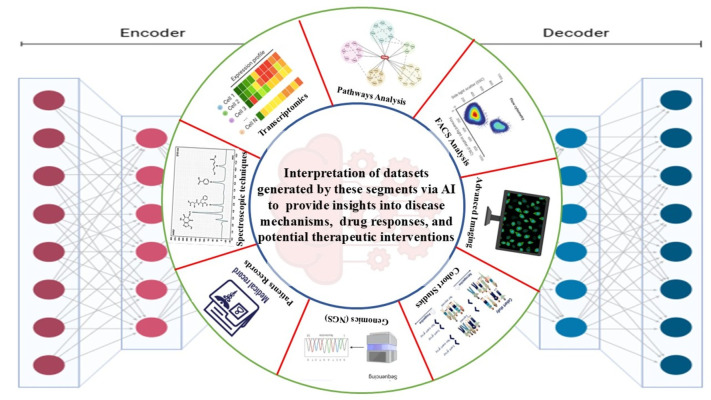
The role of AI in interpreting large datasets generated by different advanced techniques.

In conclusion, the combination of AI and in vitro models has revolutionized pulmonary research by enhancing data analysis, improving predictive modeling, optimizing drug delivery, and advancing personalized medicine. AI provides the tools needed to extract meaningful insights from complex biological data, accelerating the understanding of lung diseases and the development of effective therapies [125]. As computational power and AI algorithms continue to evolve, their integration with in vitro models will undoubtedly play an even more central role in advancing pulmonary research and improving patient outcomes. This synergy holds the potential to transform the landscape of pulmonary medicine, enabling more precise, efficient, and personalized approaches to the treatment of lung diseases (Figure 4).

## 8. Limitations, Standardization Challenges, and Future Perspectives Regarding Advanced Pulmonary Models

Despite the remarkable progress in developing advanced in vitro pulmonary models, several technical and biological limitations remain that restrict their full translational potential for respiratory disease modelling and drug discovery. While 3D culture systems and lung organoids offer significant advantages over traditional monolayer cultures, issues related to reproducibility, microenvironmental complexity, scalability and standardization continue to pose challenges for widespread adoption in therapeutic development pipelines. One of the most frequently reported limitations of organoid-based systems is batch-to-batch variability and reproducibility. Organoid formation relies heavily on the differentiation potential of pluripotent stem cells or primary tissue-derived progenitors, both of which can exhibit significant donor-to-donor variation. Differences in genetic background, epigenetic status, and culture conditions can influence cellular composition and differentiation efficiency, resulting in heterogeneity across organoid preparations. Several studies have highlighted that even minor differences in growth factor concentration or culture timing can alter lineage specification within lung organoids, ultimately affecting experimental outcomes [109,110].

Another major challenge arises from the widespread dependence on extracellular matrix (ECM) substitutes such as Matrigel, which remains the most commonly used scaffold for organoid culture. Although it provides a supportive environment for organoid growth, its poorly defined composition and lot-to-lot variability introduce significant experimental variability [142]. In addition to ECM-related challenges, the absence of vascular and immune components remains a key limitation of most current lung organoid systems [143]. Another limitation concerns scalability and throughput, particularly in the context of drug screening application. While 2D cell culture platforms enable high-throughput screening of thousands of compounds, organoid systems are significantly more labor-intensive and require specialized culture conditions. The generation of large numbers of uniform organoids remains technically challenging, limiting their application in large scale pharmaceutical screening programs [144].

Recent advances in microfluidic lung-on-chip technologies offer promising solutions to several of these limitations by introducing dynamic mechanical forces, vascular perfusions, and multi-cellular interactions into in vitro systems. Lung-on-chip platforms can replicate physiologically relevant mechanical cues such as breathing motions and fluid flow, thereby improving the physiological relevance of in vitro lung models [24]. Integration of these platforms with organoid-derived epithelial cells may enable the development of more comprehensive pulmonary models capable of recapitulating the complex disease microenvironment.

In parallel, the integration of computational modelling and AI with advanced in vitro systems is emerging as a powerful strategy to enhance predictive accuracy in respiratory research. Machine learning approaches can analyze large-scale multi-omics datasets generated from organoids and identify predictive biomarkers of disease progression or drug response. AI-driven image analysis has also been successfully applied to quantify morphological changes and cellular differentiation patterns in organoid cultures. However, the reliability of such models depends heavily on the quality and diversity of training datasets and issues such as overfitting, algorithmic bias, and lack of external validation must be carefully addressed to ensure robust predictive performance [145].

Looking forward, the next generation of pulmonary models will likely involve multi component platforms that combine organoids, microfluidic systems, engineered biomaterials, and computational analytics. Advances in vascularized organoid engineering, immune competent co-culture systems, and patient-specific stem cell technologies may significantly enhance the physiological fidelity of these models. Additionally, increasing collaboration between academic researchers, clinicians, and regulatory agencies will be critical for establishing standardization validation frameworks that enable the integration of advanced in vitro models into drug development pipelines.

## 9. Conclusions

The advancement of in vitro pulmonary disease models has revolutionized experimental respiratory research, providing physiologically relevant alternatives that more accurately capture the complexity of the human lung. Among these, 3D lung organoids stand out as powerful systems capable of recapitulating native tissue architecture, multicellular interactions, and patient-specific responses, thus bridging critical gaps between 2D and in vivo model systems. The integration of computational modelling and artificial intelligence is equally transformative, enhancing the analytical, predictive, and translational potential of biological platforms. AI-driven tools enable efficient image analysis, biomarker identification, and virtual disease simulation, complementing organoid-based experimentation with data-rich insights. Together, these technologies establish a synergistic framework that advances our understanding of pulmonary pathophysiology, accelerates drug discovery, and supports the transition toward precision and personalized medicine. Continued interdisciplinary collaboration between experimental biologists, bioengineers, and computational scientists will be essential for fully realizing the potential of these next-generation lung models in transforming pulmonary research and therapeutic development.

In summary, the concept of a translational hierarchy emphasizes that no single in vitro model is universally optimal; rather, model selection should align with specific experimental objectives. While lower-tier systems offer scalability and efficiency, higher-order models, such as organoids and lung-on-chip platforms, provide greater physiological relevance. Importantly, the integration of artificial intelligence and computational approaches across this hierarchy significantly improves data interpretation and translational potential. Collectively, a multi-model and integrative strategy is essential for advancing respiratory therapeutic development.

## Figures and Tables

**Table 1:** Comparative evaluation of in vitro pulmonary model systems for therapeutic development.

Model System	Biological Complexity	Throughput	Cost	Reproducibility	Scalability	Translational Fidelity
2D Cell Cultures	Low	Very high	Low	High	Excellent	Low
2.5D Cell cultures	Moderate	Moderate	Moderate	Moderate	Moderate	Moderate
3D Spheroids	Moderate	Moderate	Moderate	Moderate	Limited	Moderate
Lung Organoids	Very High	Low	High	Variable	Limited	Very High
Organ-on-Chip System	Very High	Very Low	Very High	Moderate	Limited	Very High
AI-Integrated Platforms	Data dependent	High	Moderate	Data dependent	High	Potentially Very High

**Table 2:** List of cell lines commercially available for modeling pulmonary diseases.

Name	Cell Type	Cell Origin/Representative Species	Disease Modelling	References
MLE-12	Cell line	Mouse alveolar epithelial cell line	ALI/ARDS/IPF	[35,42]
RLE-6TN	Cell line	Rat alveolar epithelial cell line	IPF	[43,44,45]
16HBE	Cell line	Human bronchial epithelial-like cell line	COPD/Asthma/IPF/Cystic Fibrosis	[46]
BEAS-2B	Cell line	Human bronchial epithelial cell line	COPD/Asthma/Lung Cancer	[36,47,48,49,50]
THP-1	Cell line	Human peripheral blood monocyte cell line	COPD/Asthma/Tuberculosis	[51,52]
MH-S	Cell line	Mouse alveolar macrophage cell line	Infections/Asthma/COPD	[53,54]
RAW264.7	Cell line	Mouse macrophage cell line	IPF/COPD/Asthma/Tuberculosis	[55,56]
MRC-5	Cell line	Human embryonic lung fibroblast cell line	IPF	[57,58]
HFL1	Cell line	Lung fibroblast cell line	IPF/Asthma/COPD	[59,60]
Calu-3	Cell line	Human Lung Adenocarcinoma	Viral Infections	[61]
16HBE140	Cell line	Human bronchial epithelial cell line	COPD/Cystic Fibrosis	[62]
hAELVi	Cell line	Human Alveolar Epithelial Lentivirus-Immortalized Cells	IPF/ARDS	[63]
HL-60	Cell line	Human Promyelocytic Leukemia Cells	COPD/Asthma/Tuberculosis	[64]
HMC-1	Cell line	Human Mast Cell Line	Asthma/Hypersensitivity	[65]
LADR	Cell line	Lung Adenocarcinoma Drug-Resistant Cell Line	Lung Cancer	[66]
TT1	Cell line	Transduced human type 2 carcinoma cells line	ARDS/Lung injury/IPF	[67]
NCL-H441	Cell line	Human type 2 carcinoma cell line	Cytokine-mediated lung injury/Pulmonary Edema	[68]
A549	Cell line	Human lung cancer alveolar epithelial cell line	IPF/Lung Cancer	[69,70,71]

## Data Availability

No new data were created or analyzed in this study.
